# High expression of Myosin 1g in pediatric acute lymphoblastic leukemia

**DOI:** 10.18632/oncotarget.28055

**Published:** 2021-09-14

**Authors:** Laura A. Estrada-Abreo, Leonor Rodríguez-Cruz, Yanelly Garfias-Gómez, Janeth E. Araujo-Cardenas, Gabriela Antonio-Andrés, Alfonso R. Salgado-Aguayo, Darío Orozco-Ruiz, José Refugio Torres-Nava, Juan D. Díaz-Valencia, Sara Huerta-Yépez, Genaro Patiño-López

**Affiliations:** ^1^Immunology and Proteomics Laboratory, Hospital Infantil de México Federico Gómez, México City, México; ^2^Cell Biology and Flow Cytometry Laboratory, Department of Health Sciences, Universidad Autónoma Metropolitana, Iztapalapa, México; ^3^Oncologic Diseases Research Unit, Hospital Infantil de México Federico Gómez, México City, México; ^4^Laboratory of Research on Rheumatic Diseases, National Institute of Respiratory Diseases, Ismael Cosío Villegas, México City, México; ^5^Oncology Service, Hospital Infantil Moctezuma, México City, México

**Keywords:** Myosin 1g, acute lymphoblastic leukemia, high risk, biomarker, pediatric

## Abstract

Acute Lymphoblastic Leukemia (ALL) is the most frequent cancer in pediatric population. Although the treatment has improved and almost 85% of the children are cured about 20% suffer relapse, therefore finding molecules that participate in the pathogenesis of the disease for the identification of relapse and patients at risk is an urgent unmet need. Class I myosins are molecular motors involved in membrane tension, endocytosis, phagocytosis and cell migration and recently they have been shown important for development and aggressiveness of diverse cancer types, however Myo1g an hematopoietic specific myosin has not been studied in cancer so far. We evaluated the expression of Myo1g by qRT-PCR, Immunocytochemistry and Immunofluorescence in a cohort of 133 ALL patients and correlated the expression at diagnosis and after treatment with clinical features and treatment outcomes. We found high expression levels of Myo1g in Peripheral Blood Mononuclear Cells (PBMCs) from patients with ALL at diagnosis and those levels decreased after complete remission; furthermore, we found an increase in Myo1g expression on patients with 9:22 translocation and those who relapse. This study show that Myo1g is over expressed in ALL and that may participate in the pathogenesis of the disease specially in high-risk patients.

## INTRODUCTION

Acute Leukemias constitute a heterogeneous group of malignant neoplasms characterized by the clonal proliferation of hematopoietic precursors within the bone marrow; they are originated from a malignant transformation of lymphoid or myeloid progenitor cells [1]. Leukemia is the most common type of cancer in childhood, particularly affects children under 15 years old [2], with a prevalence between the ages of 2 to 5 years. Acute Lymphoblastic Leukemia (ALL) corresponds to 78% of the cancer cases diagnosed in the pediatric population [3]. This neoplasm is classified into B-cell precursors (B-ALL) and T-cell precursors (T-ALL); the immunophenotype of B-cell precursors represents nearly 85% of the leukemia cases with the remaining 15% show a T-cell phenotype [4, 5]. Risk stratification of patients is an important component of diagnosis because it can help to decide the appropriate chemotherapy treatment and although current treatments have led to a general cure rate of more than 80% in children, however 15 to 20% of them suffer relapse. Some of the known high-risk factors for relapse are: age less than a year and older than 10 years, CNS infiltration at the time of diagnosis, T-cell phenotype, 9:22 translocation and lack of response to remission. Most relapses occur in bone marrow, however, approximately 33% of the cases have relapse to the Central Nervous System (CNS) and 7% to testicles, why those sites are the target of relapse is unknown however, an hypothesis is that the leukemic cells gain the ability to produce colonies at a distance, using a variety of migration modes to achieve a successful invasion; this cellular movement depends on structural changes at the cytoskeleton level and the participation of motor proteins, chemokines and adhesion molecules [6–9].

Class I myosins are a family of actin dependent molecular motors involved in different functions like endocytosis, exocytosis, vesicle trafficking and different aspects of cell migration [10, 11], all class I myosins associate to cell membranes through the tail domain that contains a lipid binding domain helping to fulfill their functions including generating movement in the cell [12, 13], Humans have eight genes that code for these proteins Myo1a-Myo1h and are subdivided in short tail (Myo1a, b, c, d, g, h) and long tail (Myo1e, f) [11], Myo1g is exclusively expressed in hematopoietic cells and is highly expressed in B and T lymphocytes [14, 15]. We and others have reported that Myo1g plays an important role in membrane tension, cellular rigidity and regulation of the intrinsic velocity of the cell. It has also been reported that the deficiency of this protein produces changes in structures such as filopodia and microvilli [15–19]. And recently class I myosins have been gained interest in the cancer field because some of them function as tumor suppressors and some others are over expressed in different cancers [20–22], however so far there is no indication of the direct involvement of Myo1g in cancer, therefore the aim of this study was to determine the expression levels of Myosin 1g in blood cells of patients with acute lymphoblastic leukemia and to correlate this expression with the severity of the disease and with treatment outcomes in children with ALL.

## RESULTS

### Myosin 1g is over expressed in acute lymphoblastic leukemia

**Figure 1:**
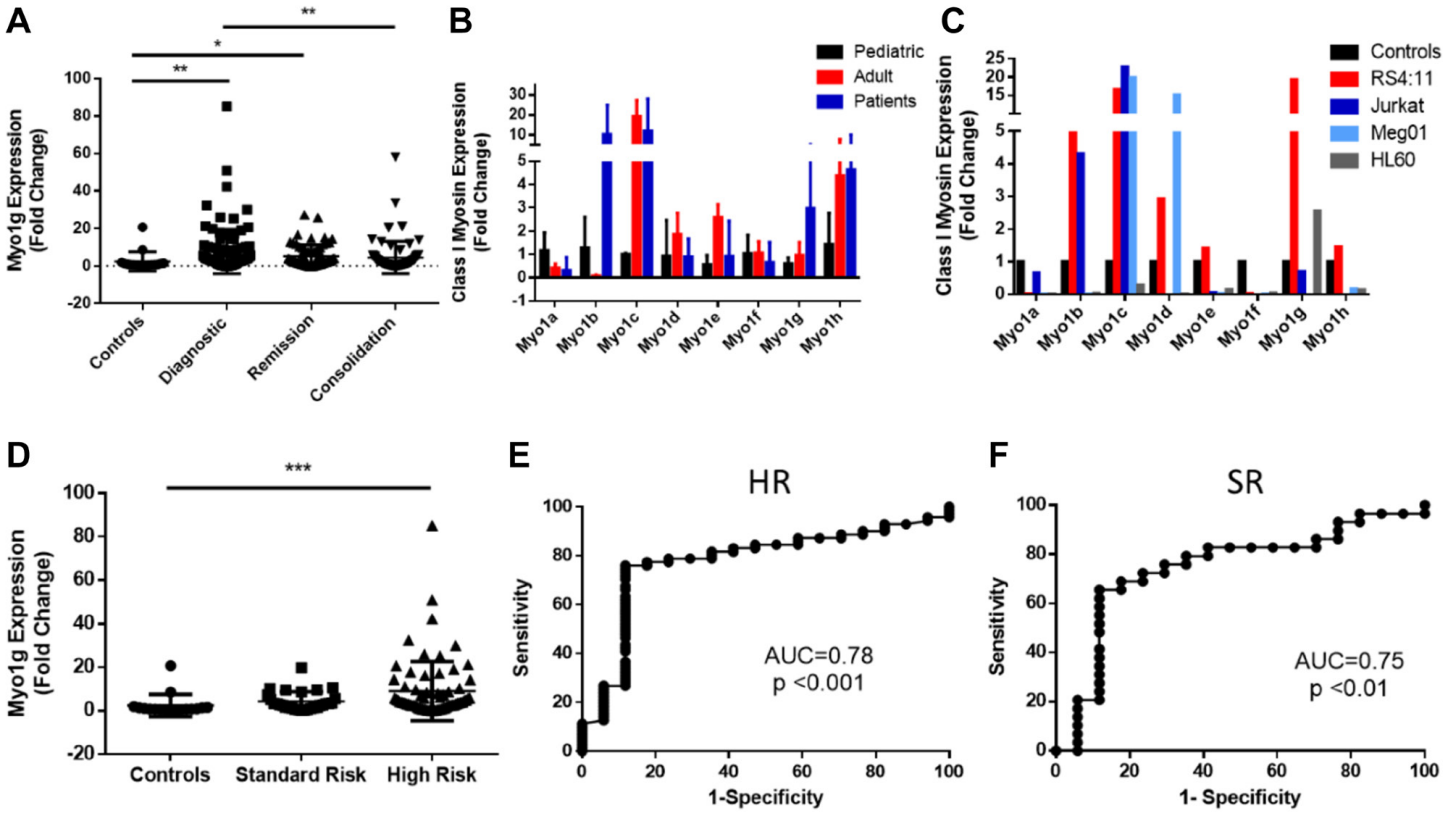
Myosin 1g is over expressed in acute lymphoblastic leukemia. (**A**) Fold Change of Myo1g mRNA expression in ALL pediatric patients (diagnostic *n* = 100, remission *n* = 68, consolidation *n* = 72) and healthy controls *n* = 17). One Way ANOVA with Dunn’s correction, Error bars represent standard deviation, ^*^ *P* < 0.05, ^**^ *P* < 0.01. (**B**) Class I myosin expression in pediatric *n* = 3 and adult *n* = 3 control PBMC cells compared to PBMCs from ALL patients *n* = 10. Error bars represent standard deviation. (**C**) Class I myosin expression in B-ALL (RS4;11), T-ALL (Jurkat), CML (Meg-01) and APL (HL-60) cell lines representative result from an experiment done in triplicates, (**D**) Myo1g expression in standard *n* = 19 and high-risk patients *n* = 71 compared to the expression in control individuals *n* = 17. (**E**) ROC curves with corresponding AUC values for high-risk patients and (**F**) standard risk patients using the level of expression in control individuals as reference.

Myosins have various functions in different cell lineages [10, 12] and recently their study has gained interest in cancer research [23–27]. To determine the expression level of Myo1g in ALL, we initially quantified the mRNA expression of Myo1g in 9 samples of peripheral blood and 9 samples of bone marrow from the same patients, subsequently we analyzed peripheral blood samples from patients, we follow them during the course of the treatment (when possible), specifically we analyzed 102 patients at diagnostic, 70 at remission and 73 at consolidation and compared those levels with 17 pediatric controls by real time PCR, the expression of GAPDH was used as internal control. We observed upregulation of Myo1g in all phases of the disease, being more significant at diagnostic with a trend to diminish at consolidation (^**^ *P* < 0.01 diagnostic, ^*^ *P* < 0.05 at remission and NS at consolidation) Figure 1A. Moreover we explored the expression of all 8 members of the class I myosin subfamily to determine which of them are upregulated in pediatric ALL, we compared levels of these Myosins in 10 hematopoietic normal pediatric individuals, 10 healthy adults and 10 patient PBMCs and found that Myo1b and Myo1g were the two Myosins more upregulated in patients Figure 1B, interestingly we found differences in myosin I expression between pediatric and adult controls, indicating that the best comparison should be pediatric individuals. We also evaluated expression of Myo1g in different cell lines, we found that Myo1g was consistently over expressed in a B-ALL cell line (RS4:11) Figure 1C. Interestingly when we classified patients according to the risk, we found that myo1g was upregulated in high-risk patients (^***^ *P* < 0.001, and was Not Significant *p* = 0.06 in Standard risk patients) Figure 1D, indicating that Myo1g could be associated with high-risk patients of pediatric ALL. To determine whether Myo1g expression could predict patients with ALL versus normal controls we generated ROC curves, we found AUC value of 0.78, *P* < 0.001 for high-risk Figure 1E and 0.75, *P* < 0.01 for standard risk patients Figure 1F, using the expression of Myo1g in the control group as reference. Taken together these data indicated that Myo1g expression is increased in ALL specially in high-risk patients.

### Myosin 1g over expression has diagnostic value in ALL

**Figure 2:**
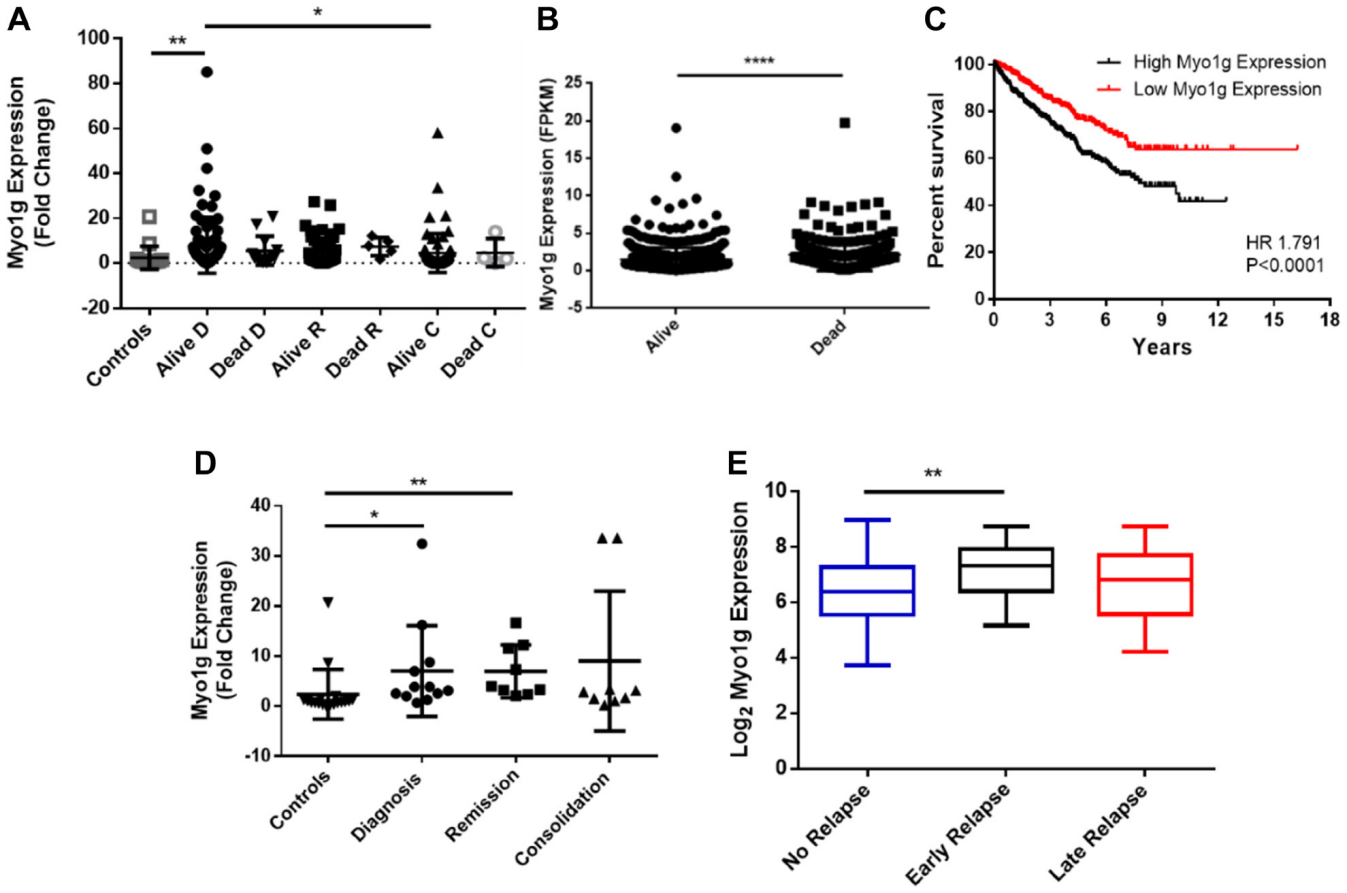
Myo1g is a potential biomarker in pediatric acute lymphoblastic leukemia. (**A**) Fold change of Myo1g expression in peripheral blood mononuclear cells (PBMC) of ALL pediatric patients alive *n* = 87 or dead *n* = 13 after diagnosis, alive *n* = 63, dead *n* = 5 at remission and alive *n* = 68, dead *n* = 4 at consolidation and controls *n* = 17. Error bars represent mean +/– SD, One-way ANOVA with Dunn’s correction ^*^ *P* < 0.05, ^**^ *P* < 0.01. (**B**) mRNA expression levels of Myo1g from TCGA data from Liver cancer patients alive *n* = 651 and death *n* = 226. Unpaired *t* test, *P* < 0.0001. (**C**) Kaplan-Meier curve of overall survival of Myo1g mRNA expression data from TCGA renal cancer data containing 877 patients, the median of Log 2 expression was used to define low (*n* = 438) and high (*n* = 439) expression (**D**) Fold Change of Myo1g expression in PBMCs from patients who relapse after Diagnostic *n* = 12, at remission *n* = 9 and at consolidation *n* = 9 compared to controls *n* = 18. Error bars represent mean +/– SD, One-way ANOVA with Dunn’s correction, ^**^ *P* < 0.01, ^*^ *P* < 0.05. (**E**) mRNA expression of Myo1g in patients with no relapse *n* = 157, early relapse *n* = 26 and late relapse *n* = 14, data from GEO GSE13576, One Way ANOVA ^**^ *P* < 0.01.

**Figure 3:**
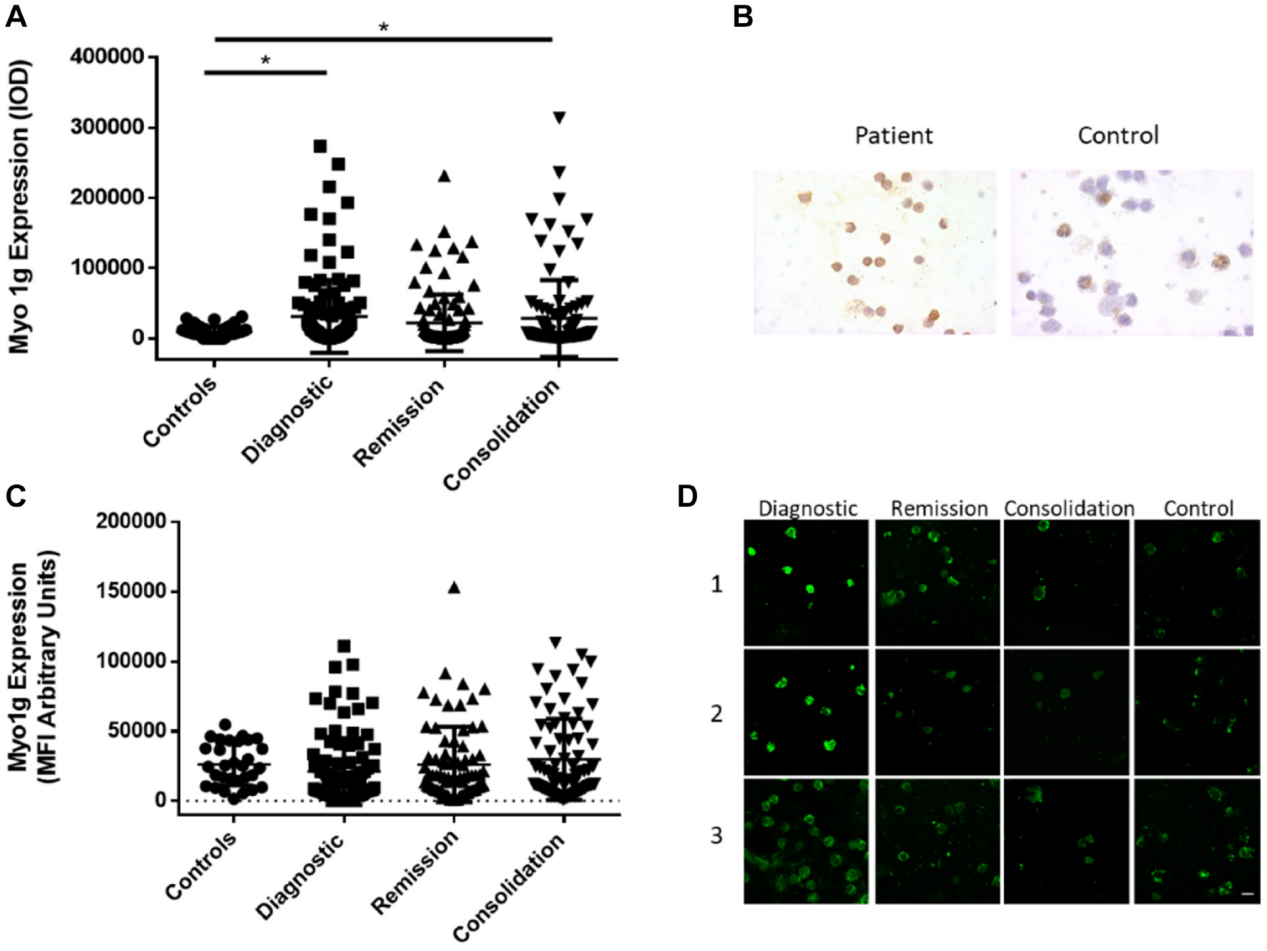
Myo1g over expression is conserved at protein level in acute lymphoblastic leukemia. (**A**) Intensity of Optical Density (IOD) of Myo1g expression in PBMCs from control individuals *n* = 61, patients at Diagnostic *n* = 117, patients at remission *n* = 105 and patients at consolidation *n* = 106. Error bars represent mean +/– SD, One-way ANOVA with Dunn’s correction, ^*^ *P* = 0 < 0.05. (**B**) Representative images of Myo1g expression at diagnosis from a patient and a control individual. (**C**) Mean fluorescence intensity of Myo1g expression in PBMCs from patients with no relapse and with relapse at Diagnostic (**D**) *n* = 93, *n* = 8 respectively, patients at remission *n* = 79 and *n* = 4 respectively and patients at consolidation *n* = 77 and *n* = 4 respectively. Error bars represent mean +/– SD, Unpaired *t* test, ^*^ *P* = 0 < 0.05. (D) Representative confocal images of Myo1g expression in 3 patients at diagnostic, remission and consolidation and 3 control individuals, bar represents 10 μm.

To determine the clinical significance of Myo1g over expression in ALL with other clinical parameters we evaluated our cohort of patients and followed the expression of Myo1g over the treatment at the remission phase and at consolidation comparing different outcomes, for this we evaluated Myo1g expression levels in PBMCs from the patients who live and died and scored the Fold Change of Myo1g on those cells. We found that patients at diagnosis have higher expression of Myo1g compared to controls ^**^ *P* < 0.01, interestingly Myo1g expression have a trend to decrease after treatment returning to levels similar to those of the controls ^*^ *P* < 0.05 Figure 2A. Unexpectedly we didn’t find obvious over expression of those patients who died at the different phases of treatment Figure 2A. To our knowledge Myo1g has not been studied as a marker in other diseases; however, TCGA data from Renal Cancer patients (857 cases) showed significant less expression of Myo1g in those patients who survived versus those who died ^****^ *P* < 0.0001 Figure 2B. Using the same dataset from TCGA we evaluated the prognostic value of Myo1g in the renal cancer patients and found a significant difference (*p* < 0.0001) indicating that high Myo1g expression correlate with poor survival Figure 2C. However, when we analyzed the prognostic value of Myo1g in our data we didn’t find significant differences (not shown) this maybe is caused by the low patient number in our cohort, further studies might test this possibility. ALL therapy has improved in the recent years however, still there are 10–15% of patients who relapse and those almost invariably will die within few years, therefore a way to identify early on those patients at risk would improve their outcome, increasing their life expectancy and event free survival, along this line we decided to evaluate if there was a correlation between Myo1g expression and patient relapse, we found that compared to controls the patients who relapse expressed higher levels of Myo1g at diagnostic ^*^ *P* < 0.05, and at remission ^**^ *P* < 0.01 but not significant difference at consolidation Figure 2D, interestingly data from a published dataset (GSE13576) which, provide data for relapse in leukemia patients showed statistical significance in the expression of Myo1g on those patients who had early relapse (*P* = 0.0008) Figure 2E. To determine the diagnostic value of Myo1g with other high risk clinical parameters we compared the expression of Myo1g in controls with patients (Supplementary Figure 1) and generated ROC curves, we found differences for patients with poor prednisone response (PPR) ^**^ *P* = 0.0017, AUC 0.792, *P* < 0.082, Patients who have no remission ^**^ *P* = 0.0016, AUC 0.812, *P* < 0.001, Patients with infiltration to SNC P = NS, AUC 0.676, *P* < 0.086 and interestingly in patients with translocation t(9;22) at diagnostic *P* < 0.05, AUC 0.889, *P* < 0.017 (Supplementary Figure 1). Taken together our results indicate that Myo1g could be used as a potential diagnostic marker, further studies will be designed to analyze this possibility. Immunocytochemistry (ICQ) is a technique of routine used in pathology laboratories in diseases such as leukemia, therefore we evaluated if Myo1g behaves similarly by ICQ and Immunofluorescence as does for mRNA expression, in normal cells Myo1g is mainly expressed at the plasma membrane [14, 15], when we evaluated Myo1g expression in the same cohort of patients, we found a significant difference between healthy controls and patients in all phases of treatment Figure 3, we observed an increased Myo1g expression by ICQ at diagnosis and consolidation ^*^ *P* < 0.05 Figure 3A, by this technique we detected membrane and cytoplasmic signal Figure 3B, again we evaluated correlation of Myo1g expression with high risk clinical parameters by ICQ compared to controls, we found ^**^ *P* < 0.01 in patients with high risk and *p* < 0.05 in patients with no remission (Supplementary Table 1), unexpectedly we did not found significant difference in Myo1g expression by immunofluorescence Figure 3C, although, we observed plasma membrane staining in normal and leukemic cells and found that leukemic cells often expressed elevated levels also at the cytoplasm and those levels continued higher after treatment Figure 3D. and evaluating Myo1g expression with high risk clinical parameters we found increased expression in patients with high risk ^***^ *P* < 0.001, patients with Translocation t(9:22) ^**^ *p* < 0.01, patients with infiltration to CNS ^***^ *P* < 0.001 and patients with no remission ^*^ *P* < 0.05 (Supplementary Table 2). Overall, our results show that Myo1g is over expressed in ALL patients and continue over expressed even early at consolidation, however, we found a trend towards basal levels in those patients who are responding well to the treatment.

## DISCUSSION

Acute Lymphoblastic Leukemia is the cancer with the highest incidence in children worldwide, although the cure rate has increased with current therapies; we still find about 20% of patients who relapse and others who do not respond to treatment. Therefore, it is necessary to identify molecules that help to better diagnose the patients, molecules that predict the course of the disease and the stratification of risk to improve the outcomes and to give more tailored treatment to the patients [28, 29]. Class I myosins recently have gained interest as potential biomarkers for different cancer types and for their participation in the development of the disease [24–26, 30]. We evaluated the expression of Myo1g an hematopoietic restricted class I myosin that has not been studied in cancer before by qPCR, Immunofluorescence and ICQ at diagnosis, after complete remission and early in consolidation. We found that Myo1g is over expressed at mRNA and to some extent at protein level in PBMCs from ALL patients and identified Myo1g as a molecule that participates in the course of the disease, we suggest that it is involved in the pathogenesis of the pediatric ALL. Although was more significant for High-Risk patients, we also detected differences in patients with infiltrations, those who suffer relapse and for those patients at high risk of death. Importantly, we used data from a previous study that used microarrays to evaluate expression of differential genes in patients at relapse, we found a statistically significant difference *P* < 0.05 between those patients who suffer early relapse versus those with no relapse on the expression of Myo1g [28] consistent with our results, from those patients who relapsed at diagnostic and at consolidation, however we need more patients and longer follow up to determine the predictive value of Myo1g at relapse. Myo1g expression levels were considerably higher ^*^ *P* = 0.0177 and ^**^ *P* < 0.05 by qRT-PCR and ICQ respectively in patients with t(9:22), those patients classically are considered as very high risk because these leukemic cells are often resistant to therapy [1, 31, 32]. Therefore, will be interesting to evaluate te predictive value of Myo1g on this subset of patients. Myo1g has been associated with cell migration and cell adhesion in B and T lymphocytes [16, 18, 33], and therefore is likely to be involved in leukemia infiltration, Importantly Myo1g deletion in mice has sown impairment of B cell migration [16] unfortunately, so far there are no studies over expressing Myo1g in B cell lines or primary B cells demonstrating and increase in cell migration, that will be subject of future work in our lab, also over expressing Myo1g and transferring those cells into nude mice to induce tumors and evaluate their ability to infiltrate different tissues will be an interesting approach. Myo1g in normal conditions is exclusively expressed in hematopoietic cells however, Myo1g over expression (at mRNA level) is of bad prognostic in Renal Cancer in TCGA data, indeed high expression of Myo1g indicates poor survival, similar trend was observed in our patients however, we didn’t find significant differences in patient survival, but those patients who expressed high levels of Myo1g (at protein and mRNA level) had in general poor survival. One intriguing feature of the Myo1g over expression is that there was no significant decrease in the expression as the patients continued their treatment (consolidation); one possibility is that longer follow up is required and that Myo1g expression will return to base line levels after long maintenance period. Myo1g expression was similar in peripheral blood and bone marrow samples, this fact allowed us to continue the studies with PBMCs, a routine visit with a blood sample could help to evaluate the risk in the children because we detected elevated levels of Myo1g at diagnosis; therefore, maybe implementing regular test of Myo1g in children at risk could help to detect cancerous cells at an early stage.

Class I myosins recently have gained interest in cancer research because some are over expressed and contribute to increase disease severity, for example Myo1b in head and neck squamous cell carcinoma (HNSCC) promotes cell migration and lymph node metastasis, Myo1e in breast cancer promotes proliferation and tumor de differentiation [24, 30], some others are down regulated like Myo1a in colon cancer were seems to function as a tumor suppressor [25, 26]. The analysis of Myo1g expression in Leukemia showed that is a molecule that participates in the development of the pediatric ALL and together with the clinical characteristics could strengthen the initial diagnosis, although we know that the diagnosis of ALL is confirmed by the bone marrow aspirate, an extra indication in PBMCs could contribute to the correct patient stratification, further studies are necessary to define and to identify the underlying biochemical and functional mechanisms of the over expression of this myosin in the leukemic cells.

In summary, this study illustrates that Myo1g expression might participate in the pathogenesis of the disease specially in high-risk patients of acute lymphoblastic leukemia and patients with translocation t(9:22).

## MATERIALS AND METHODS

### Patients and samples

All human samples used in this study were authorized by written informed consent and approved by the Research Ethics and Bio safety Committee of the Hospital Infantil de Mexico, Federico Gómez and Hospital Pediátrico Moctezuma.

Peripheral blood was obtained from a cohort of 133 patients with acute lymphoblastic leukemia (ALL) and 10 samples of bone marrow from the same patients admitted from September 2015-July 2018. The clinical data are summarized in Table 1. Samples were obtained in EDTA tubes, at diagnosis and at two different stages of treatment: complete remission (in average Day 28) and one month after complete remission (consolidation). None of the patients had received treatment before diagnostic.

**Table 1:** Clinical characteristics of the study population

Patients ALL	133
**Age**	Range (7 months to 16 years)
**Gender *n*, (%)**	52, (39) F/81 (61) M
**Risk stratification (ALL patients)**	HR 93/SR 39
**ALL Immunophenotype**	Pro - B 15
	Pre - B 87
	T 9
	B 17
**Deaths ALL *n*, (%)**	22, (16.5)
**Infiltration ALL *n*, (%)**	22, (16.5)
**Translocation 9:22 *n*, (%)**	5, (3.75)
**Relapse (%)**	12, (9.02)
**No remission *n*, (%)**	15, (19.54)
**Controls**	*n* = 61
**Age**	Range (1–15 years)

We included 60 no leukemia children as a control cohort. Informed consent was granted from all participants. The control children are in an age range of 1-15 years old, and during their participation in the study they did not present alterations in hematological values.

### Isolation of PBMCs

Peripheral blood mononuclear cells were isolated by density gradient with Lymphoprep (Axis Shield), following the manufacturer’s instructions. After this the samples were divided in two parts: one was stored in Trizol at –80°C for mRNA extraction and the other was used for immunofluorescence and immunocytochemistry. Before staining, the cells were fixed in 4% paraformaldehyde on slides and then washed with PBS 1X.

### RNA isolation, reverse transcription and quantitative real-time PCR

RNA was extracted from PBMCs using the RNeasy mini kit (QIAGEN), and reverse transcribed into cDNA using the Quantitect Reverse Transcription kit (QIAGEN). Myosin 1G expression was measured by quantitative PCR using the Agilent Mx3005 P thermocycler, Universal Probe Library (UPL) (Roche) with a specific primer set for myosin1G. Glyceraldehyde 3-phosphate dehydrogenase (GAPDH) gene was used as the internal control; for amplification we used the Light Cycler 480 (Roche) master mix, amplification was carried out at 95°C/10 s, 56–59°C/30 s, 72°C/11 s for 45 cycles. Fold Change values of gene expression were calculated with the 2^-ΔΔCt^ method using the average from triplicate measurements.

### Immunofluorescence microscopy

Cells were washed in 0.1% PBS / Tween 20 for 40 min. Samples were blocked with 2% Pig Serum (PS) and 0.5% Triton X-100 in a humidity chamber for 1 h 40 min, then washed twice for 2 min. Samples were incubated with the primary antibody for myosin 1G, previously described [14] at room temperature overnight at a 1:100 dilution. After five washes with 0.1% PS in PBS/Tween 20, samples were incubated for 1 h at room temperature with goat anti-rabbit IgG Alexa 647 (Jackson ImmunoResearch) at 1:200 dilution; finally, cells were washed twice in PBS/Tween 20. Coverslips were mounted on glass slides using vectashield mounting medium with DAPI (Vector Labs).

To image the stained cells, we used an Olympus FV1000 confocal microscope. We captured no less than 5 micrographs of the median plane of the cells at 60X using identical settings for each capture, making sure that no saturated pixels were detected. We quantified fluorescence intensity from 50 to 100 cells per patient at diagnosis and in the different phases of treatment (remission and consolidation). Images were analyzed using Fiji, Image J software (NIH); specifically, we draw a line in the periphery of each cell to measure the mean fluorescence intensity (MFI) in each cell. Results were expressed as the average of MFI for each patient using arbitrary units for the quantification.

### Immunocytochemistry

The slides were incubated with antibodies for myosin 1G [14] or normal rabbit IgG’s to identify nonspecific binding (Normal Rabbit Serum, Santa Cruz Biotechnology). Cells were hydrated with PBS 1X for 5 minutes before antigen recovery with sodium citrate (0.01M, pH 6.0) for 15 min at 90°C. Samples were washed twice with PBS 1X. Endogenous peroxidase activity was eliminated with two washes of 15 min with methanol and 3% hydrogen peroxide. We used the same conditions for blocking and primary antibody incubation as in immunofluorescence assays, but anti-Myo1g was used at a 1:750 dilution. Cells were incubated with ImmPRESS anti-Rabbit-HRP (Vector Laboratories) for 10 min at room temperature, then washed with PBS and incubated with streptavidin for 10 min. Color was developed incubating the sample with the chromogen Diaminobenzidine (Dako) for 1 min; the reaction was stopped with water and samples were counterstained with hematoxylin for 1s Samples were then dehydrated under the following conditions: distilled water, 70% ethanol, 90% ethanol, 100% ethanol and xylene; finally, glass slides were covered with resin. Cells were visualized using an Olympus BX-40 microscope, intensity from 50 to 100 cells per patient at diagnosis and in the different phases of treatment was measured to obtain the IOD (Intensity of Optical Density), using the software Image-Pro Plus V. 6.0. The brown colour staining of the cellular periphery was quantified and all values from one patient per treatment phase were averaged. Results were expressed as the average of the quantified pixels (IOD) in each patient.

### Statistical analyses

Statistical tests were performed with GraphPad Prism Software (version 6.01, La Jolla, CA, USA). Comparison between 2 groups was evaluated by unpaired *t*-test or Mann Whitney test (for variables with no normal distribution). For multiple comparisons, we performed one-way ANOVA and Kruskal-Wallis with Dunn’s correction for variables with non-normal distribution. The suitability of Myo1g as a biomarker that discriminates ALL patients and normal subjects was evaluated by calculating the area under the curve (AUC) using SPSS version 20. Kaplan Meier analysis was performed to estimate the survival function from lifetime data. Statistical significance was assessed by Log Rank test. Unless otherwise stated results are shown mean +/– SD. *P* values < 0.05 were considered as statistically significant.

## SUPPLEMENTARY MATERIALS


