# Supplementary material for: High expression of Myosin 1g in pediatric acute lymphoblastic leukemia
Source: Oncotarget. 2021 Sep 14;12(19):1937–45. doi: 10.18632/oncotarget.28055 (PMC8448507; doi:10.18632/oncotarget.28055)
Supplement: Supplementary file 1 [file oncotarget-12-1937-s001.pdf]

High expression of Myosin 1g in pediatric acute lymphoblastic leukemia

SUPPLEMENTARY MATERIALS

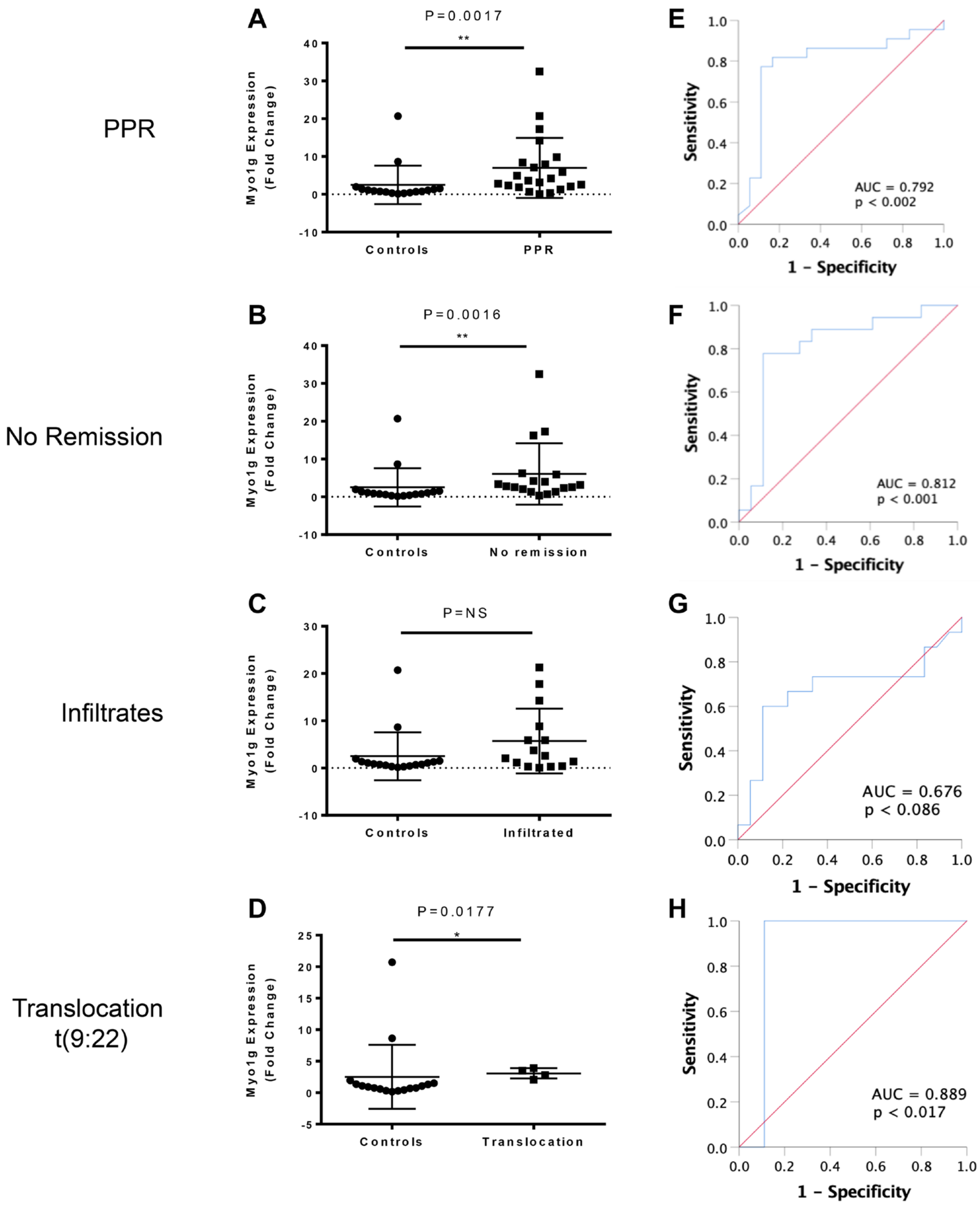

Supplementary Figure 1: Myo1g expression correlates with high-risk parameters in ALL patients and ROC curves for. Myo1g expression in Poor Prednisone Response patients (**A**, **E**), patients with no Remission (**B**, **F**) patients with infiltration to CNS (**C**, **G**) and patients with translocation t (9:22) (**D**, **H**), *P* values indicated in each graph, Mann Whitney test (A–D) and ROC curves with AUC and *P* values for each parameter (E–H).

Supplementary Table 1: High risk clinical criteria and Myo1g expression by ICQ

	n	Group	*p*
Control	62		
	34	DG/Ctl	0.286
ALL-SR	32	RM/Ctl	0.6354
	32	Cons/Ctl	0.907
	87	DG/Ctl	**0.006**
ALL-HR	75	RM/Ctl	0.9179
	75	Cons/Ctl	0.4534
	17	DG/Ctl	0.2471
Deaths	5	RM/Ctl	0.8081
	5	Cons/Ctl	0.0659
	5	DG/Ctl	0.2981
Translocation t(9:22)	4	RM/Ctl	0.4272
	4	Cons/Ctl	0.1478
	11	DG/Ctl	0.8609
Relapse	9	RM/Ctl	0.3364
	9	Cons/Ctl	0.7294
	21	DG/Ctl	0.067
Infiltration	17	RM/Ctl	0.5488
	17	Cons/Ctl	0.5568
	24	DG/Ctl	**0.0141**
No Remission	16	RM/Ctl	0.5193
	16	Cons/Ctl	0.2655

P values from the expression of Myosin 1G (Immunocytochemistry (IOD)) with clinical characteristics of the patient population compared to pediatric controls. The values were calculated using the Mann Whitney *U* test and unpaired *t*-test. Abbreviations: DG: Diagnosis; RM: Remission; Cons: Consolidation; Ctl: Control.

Supplementary Table 2: High risk clinical criteria and Myo1g expression by immunofluorescence

	n	**Group**	***p***
Control	30		
	25	DG/Ctl	0.2394
ALL-SR	23	RM/Ctl	0.4443
	23	Cons/Ctl	0.2797
	78	DG/Ctl	**0.0005**
ALL-HR	66	RM/Ctl	0.051
	63	Cons/Ctl	0.3191
	21	DG/Ctl	0.3127
Deaths	5	RM/Ctl	0.3129
	5	Cons/Ctl	0.7011
	4	DG/Ctl	**0.0048**
Translocation t(9:22)	2	RM/Ctl	0.2559
	3	Cons/Ctl	0.2315
	7	DG/Ctl	0.1012
Relapse	4	RM/Ctl	0.8971
	4	Cons/Ctl	0.5424
	19	DG/Ctl	**0.0008**
Infiltration	14	RM/Ctl	0.1052
	14	Cons/Ctl	0.3357
	16	DG/Ctl	**0.0079**
No remission	7	RM/Ctl	0.3652
	8	Cons/Ctl	**0.0445**

P values from the expression of Myosin 1G (Immunofluorescence (MFI)) with clinical characteristics of the patient population compared to pediatric controls. The values were calculated using the Mann Whitney *U* test and unpaired *t*-test. Abbreviations: DG: Diagnosis; RM: Remission; Cons: Consolidation; Ctl: Control.
